# Selenium Protects Neonates against Neurotoxicity from Prenatal Exposure to Manganese

**DOI:** 10.1371/journal.pone.0086611

**Published:** 2014-01-22

**Authors:** Xin Yang, YiXiao Bao, HuanHuan Fu, LuanLuan Li, TianHong Ren, XiaoDan Yu

**Affiliations:** 1 MOE-Shanghai Key Lab of Children’s Environmental Health, Xinhua Hospital Affiliated to Shanghai Jiao Tong University School of Medicine, Shanghai, China; 2 Department of Pediatrics, Xinhua Hospital Affiliated to Shanghai Jiao Tong University School of Medicine, Shanghai, China; Hôpital Robert Debré, France

## Abstract

Manganese (Mn) exposure can affect brain development. Whether Selenium (Se) can protect neonates against neurotoxicity from Mn exposure remains unclear. We investigated this issue in 933 mother-newborn pairs in Shanghai, China, from 2008 through 2009. Umbilical cord serum concentrations of Mn and Se were measured and Neonatal Behavioral Neurological Assessment (NBNA) tests were conducted. The scores <37 were defined as the low NBNA. The median concentrations of cord serum Mn and Se were 4.0 µg/L and 63.1 µg/L, respectively. After adjusting for potential confounders, the interaction between Se and Mn was observed. Cord blood Mn levels had different effects on NBNA scores stratified by different cord blood Se levels. With Se<P50 (<63.1 µg/L), Mn was negatively associated with NBNA scores (adjusted ß = −1.1, 95% CI: −1.3 to −0.9, p<0.001) and a higher cord blood Mn level increased the risk of low NBNA (adjusted OR = 5.7, 95% CI: 2.8 to 11.5, p<0.001). However, the adverse effect of Mn was reduced with Se≥P50 (≥63.1 µg/L) (NBNA: adjusted ß = 0.1, 95% CI: −0.3 to 0.5, p = 0.746; Low NBNA: adjusted OR = 4.5, 95% CI: 0.4 to 46.7, p = 0.205). Furthermore, the high Mn exposure group with a low Se level [Mn≥P75 (9.1 µg/L) and Se<P50 (63.1 µg/L)] had much lower NBNA scores than that of high Mn exposure group with a high Se level [Mn≥P75 (9.1 µg/L) and Se≥P50 (63.1 µg/L)] (38.0±1.6 & 39.5±0.9, p<0.001). Mn/Se ratio and NBNA scores were moderately correlated (r = −0.41, p<0.001). Our findings suggest that Se has a protective effect on neonates’ brain development against neurotoxicity from prenatal exposure to Mn. Se supplementation should be considered during pregnancy, especially in areas with low natural Se.

## Introduction

Manganese (Mn) is an essential element for brain growth and metabolism, but too much of it has the potential to cause neurotoxicity [1]. Research indicates that excessive and prolonged occupational exposure to Mn is associated with irreversible neurodegenerative disorders resembling idiopathic Parkinson disease [2]. The neurobehavioral effects of Mn are of current interest, especially when exposure occurs in the earlier stages of brain development. Fetuses are generally considered to be highly susceptible to environmental toxins. One research in Taiwan suggests that mother exposure to Mn-containing fuel from motor vehicles may result in elevated Mn levels in the fetus [3]. Prenatal Mn levels have been linked to childhood abnormal behaviors, such as more impulsive, inattentive, aggressive, defiant, disobedient, destructive, and hyperactive [4].

The neurotoxicity from Mn is mediated, at least in part, by oxidative stress [5]. Antioxidant treatment is an effective method against the toxic effects of Mn in the central nervous system. Several antioxidants have been shown to prevent the neurotoxicity from Mn, such as Trolox [6], N-acetylcysteine [7] and Diethyl-2-phenyl-2-tellurophenyl vinylphosphonate (DPTVP) [8].

Selenium (Se), is an essential component of at least 25 human selenoproteins, most of which are involved in oxidative stress protection or maintaining cellular redox balance, such as thioredoxin reductases (TRxR), glutathione peroxidases (GPx) and possibly selenoprotein P (SeP) [9]. An animal study demonstrates that ebselen, a seleno-organic compound, can inhibit Mn-induced ROS generation and is efficacious in reducing Mn-induced neuroinflammation, oxidative stress and locomotor activity impairments [10]. However, few data are available about the protective effect of Se against Mn-related neurobehavioral dysfunctions in early neurodevelopment in human. We conducted an epidemiological study in Shanghai, China to explore the potential protective effects of prenatal Se levels against Mn-induced neurotoxicity during neonate neurobehavioral development.

## Methods

### Study Protocol

We recruited 933 healthy pregnant women about to deliver a term singleton infant (37–42 weeks of gestation) in 10 maternity hospitals in Shanghai, China, from 2008 to 2009. Infants were included after birth. The study was approved by the Medical Ethics Committee of Shanghai Xinhua Hospital, affiliated with the School of Medicine, Shanghai Jiao Tong University. We had obtained written informed consent from the next of kin, caretakers, or guardians on the behalf of the minors/children participants involved in our study.

After written consent forms were signed, face-to-face interviews were conducted to collect information on their socio-demographic characteristics. Neonatal Behavioral Neurological Assessment (NBNA) was administered when the infants were 3 days old as previously described [11]. NBNA assesses functional abilities, most reflexes and responses, and stability of behavioral status during the examination. It consists of five clusters: behavior (six items), passive tone (four items), active tone (four items), primary reflexes (three items), and general assessment (three items). Each item has three levels (0, 1 and 2). Twenty items can have a maximal total score of 40. NBNA were conducted by ten examiners who were rigorously trained and certified by Professor Bao who introduced the Chinese NBNA [12]. In addition, we had let them administer NBNA in a same group of neonates and there are no significant difference among their test results, which eliminated inter-observer variation. These examiners were blinded to the prenatal Mn and Se levels.

Umbilical cord blood was collected and serum was separated. All the samples were immediately frozen in −40°C and shipped in batches to the central laboratory. Serum Mn and Se concentrations were measured by Inductively Coupled Plasma Mass Spectrometry (ICP-MS) (7500 CE, Agilent) as described previously [13]. The limit of detection (LOD) was 0.13 µg/L for Mn and 1.62 µg/L for Se. Values below LOD were imputed using the default value of 1/2 LOD.

### Statistical Analysis

**Figure 1:**
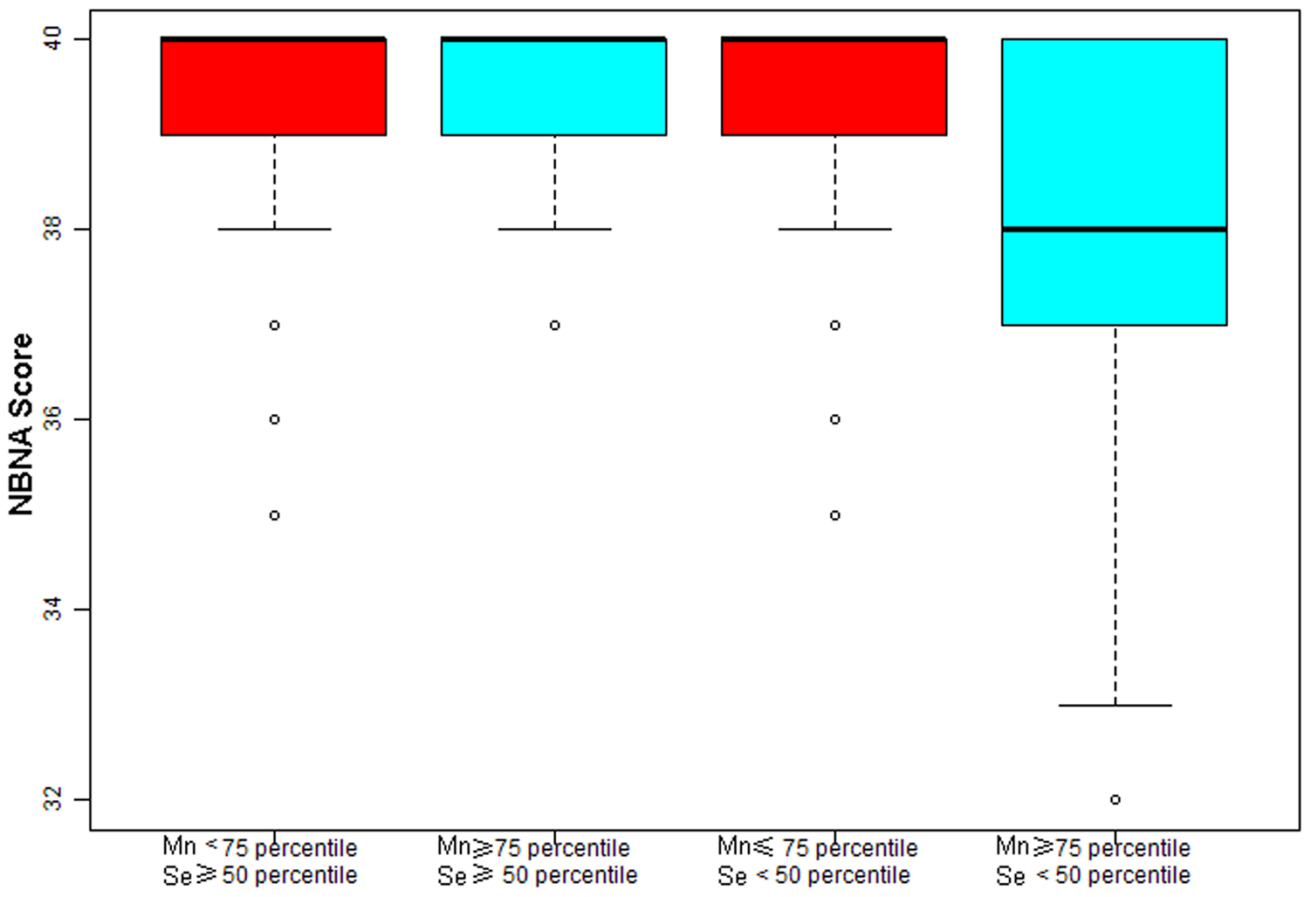
Se protects neonates against neurotoxicity from high Mn exposure. High Mn exposure population with low Se level (Mn≥9.1 µg/L and Se<63.1 µg/L) had much lower NBNA score (38.0±1.6 & 39.5±0.9) than that of high Mn exposure population with high Se level (Mn≥9.1 µg/L and Se≥63.1 µg/L) (P<0.001). Adjust for: maternal age, maternal education, paternal education, paternal education, maternal occupation, paternal occupation, family incomes, gestational age, birth weight and gender.

**Figure 2:**
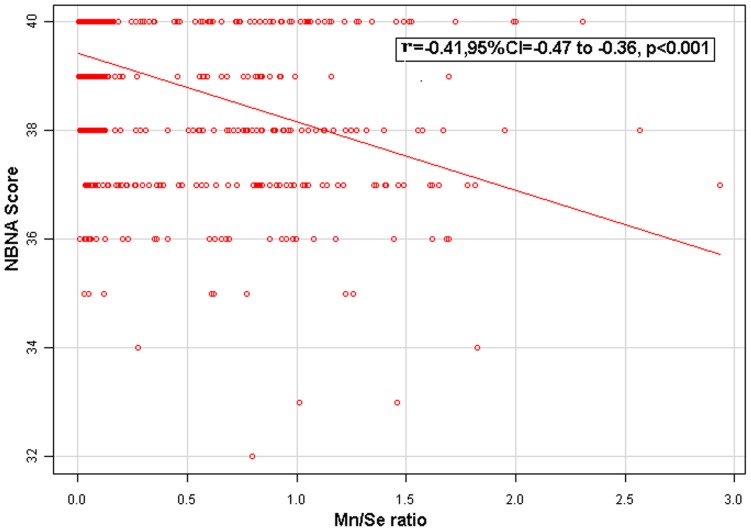
The relationship between Mn/Se ratio and NBNA score. Adjust for: maternal age, maternal education, paternal education, paternal education, maternal occupation, paternal occupation, family incomes, gestational age, birth weight and gender.

Cord serum concentrations of Mn and Se were expressed as µg/L. We first examined the distribution of NBNA score and the level of Mn and Se. The distribution of cord serum concentrations of Mn and Se were strongly skewed toward the left. Thus, we performed log10 (LgMn and LgSe) transformation before analysis. Participants were divided into four groups based on quartiles of LgMn and LgSe (Table 1). We then performed univariate analysis and multiple regression analysis to estimate the independent relationship between LgMn concentrations by LgSe stratification and mean NBNA score, and low NBNA (Table 2, Table 3). Analysis of Variance (ANOVA) was used to assess the relationship between LgMn concentrations by LgSe stratification and NBNA score (Table 4 and Figure 1). Correlation between the Mn/Se ratio and NBNA scores was analysed (Figure 2). All analyses were performed using Empower (R) (www.empowerstats.com, X&Y solutions, inc. Boston MA) and R packages (http://www.R-project.org).

**Table 1:** Factors associated with NBNA score.

		NBNA Score		Low NBNA*	
		ß (95%CI)	p-value	OR(95%CI)	p-value
**Lg Mn** **quartiles**					
<P25(Mn<2.7 µg/L)		0		1.0	
P25-50(Mn: 2.7–4.0 µg/L)		0.1 (−0.1, 0.3)	0.482	1.0 (0.3, 4.1)	0.985
P50-75(Mn: 4.1−9.0 µg/L)		0.0 (−0.2, 0.2)	0.961	1.7 (0.5, 5.9)	0.396
≥P75(Mn≥9.1 µg/L)		−1.2 (−1.4, −1.0)	<0.001	8.0 (2.8, 23.0)	<0.001
**Lg Se** **quartiles**					
<P25(LgSe<1.7 µg/L)		0		1.0	
P25-50(LgSe: 1.7–1.8 µg/L)		0.9 (0.7, 1.1)	<0.001	0.23 (0.14, 0.38)	<0.001
P50-75(LgSe: 1.8–1.9 µg/L)		1.2 (1.0, 1.4)	<0.001	0.10 (0.05, 0.20)	<0.001
≥P75(LgSe≥1.9 µg/L)		1.1 (0.9, 1.4)	<0.001	0.10 (0.05, 0.22)	<0.001
**Maternal age (years)**		0.0 (−0.1, 0.0)	0.002	1.0 (1.0, 1.1)	0.457
**Gestational age (days)**		0.0 (0.0, 0.0)	0.265	1.0 (1.0, 1.0)	0.852
**Birth weight**		0.0(0.0, −0.0)	0.192	1.0 (1.0,1.0)	0.970
**Gender**					
Male		0		1.0	
Female		−0.1 (−0.3, 0.1)	0.295	1.1 (0.6, 2.1)	0.687
**Household incomes(**Yuan/m/per person**)**					
<2000	0			1.0	
2000–5000	−0.1 (−0.3, 0.1)		0.407	1.0 (0.5, 2.1)	0.927
>5000	−0.5 (−0.7, −0.2)		<0.001	1.9 (0.9, 4.0)	0.100
**Maternal education**					
Middle school or lower	0			1.0	
High scool	−0.1 (−0.4, 0.1)		0.295	1.6 (0.7, 3.9)	0.313
Bachelor degree	−0.2 (−0.3, 0.0)		0.103	1.4 (0.6, 2.9)	0.446
Higher than Bachelor degree	−0.7 (−1.1, −0.3)		0.001	4.9 (1.7, 14.0)	0.003
**Paternal education**					
Middle school or lower	0			1.0	
High scool	0.1 (−0.2, 0.3)		0.579	0.9 (0.3, 2.6)	0.805
Bachelor degree	−0.3 (−0.5, −0.1)		0.006	1.7 (0.8, 3.8)	0.175
Higher than Bachelor degree	−0.5 (−0.9, −0.2)		0.004	4.6 (1.6, 13.0)	0.004
**Maternal occupation**					
White collar	0			1.0	
Technician	0.0 (−0.3, 0.3)		0.912	1.2 (0.4, 3.8)	0.793
Blue collar	−0.2 (−0.4, 0.1)		0.133	1.7 (0.6, 4.5)	0.313
Housewife	−0.6 (−1.3, 0.2)		0.175	3.3 (0.3, 31.0)	0.302
**Paternal occupation**					
White collar	0			1.0	
Technician	0.3 (0.0, 0.6)		0.032	0.7 (0.2, 2.3)	0.522
Blue collar	0.1 (−0.1, 0.3)		0.467	1.0 (0.5, 2.2)	0.999
Unemployed	−0.2 (−0.6, 0.2)		0.265	1.3 (0.4, 4.6)	0.700

* low NBNA: NBNA Score<37.

**Table 2:** Unadjusted stratified analysis of the effect of prenatal Mn on NBNA by different Se level.

			NBNA Score		Low NBNA *	
	LgSe	N	ß (95%CI)	p-value	OR(95%CI)	p-value
LgMn(ug/L)	<P25	263	−1.0(−1.3, −0.6)	<0.001	3.9 (2.2, 7.0)	<0.001
	P25–50	249	−0.8(−1.0, −0.5)	<0.001	8.9 (3.9, 20.5)	<0.001
	P50–75	231	−0.2(−0.6, 0.2)	0.449	1.1 (0.7, 2.5)	0.402
	≥P75	190	0.2(−0.3, 0.7)	0.474	0.4 (0.03,5.0)	0.483

* low NBNA: NBNA Score<37.

**Table 3:** Adjusted stratified analysis of the effect of prenatal Mn on NBNA by different Se level.

		NBNA Score *		Low NBNA *	
		ß (95%CI)	p-value	OR (95%CI)	p-value
Lg Mn (ug/L)	LgSe≤P50	−1.1(−1.3, −0.9)	<0.001	5.7 (2.8, 11.5)	<0.001
	LgSe>P50	0.1(−0.3, 0.5)	0.746	4.5 (0.4, 46.7)	0.205

* Adjust for: maternal age, maternal education, paternal education, paternal education, maternal occupation, paternal occupation, family incomes, gestational age, birth weight and gender.

**Table 4:** Se protects neonates against neurotoxicity from high Mn exposure.

	LgMn≥P75		LgMn<P75	
	LgSe<P50	LgSe≥P50	LgSe<P50	LgSe≥P50
N	206	39	310	378
NBNAScore	38.0±1.6	39.5±0.9	39.3±1.0	39.5±0.9
P value	<0.001		>0.05	
	LgMn≥P50		LgMn<P50	
	LgSe<P50	LgSe≥P50	LgSe<P50	LgSe≥P50
N	285	171	227	250
NBNAScore	38.3±1.6	39.5±0.9	39.4±0.9	39.5±0.8
P value	<0.001		>0.05	

Adjust for: maternal age, maternal education, paternal education, paternal education, maternal occupation, paternal occupation, family incomes, gestational age, birth weight and gender.

## Results

In total, 933 term newborns (494 males and 439 females) and their mothers were recruited. The median serum concentrations of Mn and Se were 4.0 µg/L and 63.1 µg/L, respectively. NBNA score was associated with maternal age, household income, maternal education, paternal education and paternal occupation. Low NBNA was associated with maternal education and paternal education. A high level of Mn (≥75th percentile) was associated with a lower NBNA score (ß = −1.2, 95% CI: −1.4 to −1.0, p<0.001) and a higher risk of low NBNA (OR = 8.0, 95% CI: 2.8 to 23.0, p<0.001) (Table 1).

Table 2 shows the interaction between Se and Mn without adjustment for confounders. Cord blood Mn exposure stratified by different Se levels had different effects on NBNA score. After adjusting for potential confounders, the interaction still existed (Table 3). With LgSe<P50 (Se<63.1 µg/L), Mn was negatively associated with NBNA scores (adjusted ß = −1.1, 95% CI: −1.3 to −0.9, p<0.001) and a higher Mn level increased the risk of low NBNA (adjusted OR = 5.7, 95% CI: 2.8 to 11.5, p<0.001). However, the adverse effect was reduced with LgSe≥P50 (Se≥63.1 µg/L) (NBNA: adjusted ß = 0.1, 95% CI: −0.3 to 0.5, p = 0.746; Low NBNA: adjusted OR = 4.5, 95% CI: 0.4 to 46.7, p = 0.205).

Only when LgMn>P75, the level of Mn was associated with a lower NBNA score (p<0.001) and a higher risk of low NBNA (p<0.001) (Table 1). Thus, we used “LgMn≥P75, LgMn<P75” to compare the NBNA score by different Se levels. The results show that a high Mn exposure group with a low Se level (Mn≥9.1 µg/L and Se<63.1 µg/L) had much lower NBNA scores (38.0±1.6 & 39.5±0.9) than that of a high Mn exposure group with a high Se level (Mn≥9.1 µg/L and Se≥63.1 µg/L) (p<0.001) (Table 4, Figure 1). Besides, we also used “LgMn≥P50, LgMn<P50” to compare the NBNA score (Table 4). The result was consistent with the “LgMn≥P75, LgMn<P75”. In addition, Mn/Se ratio was correlated with NBNA scores (r = −0.41, p<0.001) (Figure 2).

## Discussion

Our research had a relative large study population which provided statistical power to detect interactions between Se and Mn. This is the first epidemiological study of the protective effects of prenatal Se levels against Mn-induced neurotoxicity in the early stages of life.

Cord serum levels of elements are good indicators of *in utero* exposure [14]. The median level of cord serum Mn (4.0 µg/L) in our study was similar to the level found in Brazil (3.5 µg/L) [15] but higher than the level reported in Japan (males: 1.63 µg/L, females: 1.68 µg/L) [16]. Meanwhile, the median cord serum Se level in our study (63.1 µg/L) was similar to that reported in Baltimore (69 µg/L) [17], but higher than Christchurch (36.3 µg/L, a selenium deficient area) [18] and lower than Iran (124.8 µg/L) [19].

Neonatal self-regulation behaviors were the best predictors of infant development and intelligence. Furthermore, neonatal behavioral neurological development assessment could be a useful tool to observe behaviors related to later development in healthy infants [20]. Bao et al. [12] created the NBNA based on the method used by Brazelton and Amiel-Tison for behavioral neurological measurement in newborns as well as their own experience, and subsequently validated it in 714 normal newborns in 12 provinces of China. Repeated measurements in children showed the stability and reliability of the NBNA, which were not influenced by geographic locations [21]. The distribution of NBNA scores in our study was similar to those obtained in other cities in China [12], [21].

In the present study, we discovered a strange phenomenon which educated parents were likely to have kind with low NBNA. We speculate that this maybe due to that educated parents are relatively older in our study population and maternal age is negatively associated with NBNA score. Thus, we made age as a confounding factors and found the effect of educated parents on NBNA was eliminated.

The developing nervous system is a prime target for the disrupting effects of Mn. Mn crosses the placenta to the fetus via active transport mechanisms [22], then enters the brain across the blood brain barrier [23]. It has been shown that maternal exposure to high levels of Mn (10mg/ml) can improve the Mn levels of the pups’ corticals approximately 2.5 times than those of the control rats [24]. In the brain, the striatum, globus pallidus, and substantia nigra have a propensity to accumulate Mn and the mitochondria is primarily targets on a cellular level in both neurons and astrocytes [5]. High-doses of Mn can inhibit mitochondria respiratory chain complexes and induce the production of reactive oxygen species (ROS) [25], including superoxide, hydrogen peroxide, and hydroxyl radical [26]. In vitro study demonstrates that exposure to Mn^2+^ can produce dose-dependent increases of ROS in striatum [27] and oxidative stress in the striatum is associated with impairment of motor activity [28]. In other words, oxidative stress is a very important mechanism for Mn-induced neurotoxicity. There are also several other mechanisms involved in the neurotoxic effects of Mn, such as energy failure [29], glutamate excitotoxicity [30], dysregulation of dopamine release [31] and disruption of iron homeostasis [32].

Excess Mn exposure can exhibit an adverse effect on early neurodevelopment. We found that neonatal neurobehavioral development was damaged and the risk of low NBNA was increased with higer levels of cord serum Mn. This is consistent with previous studies [4], [33].

Our study also demonstrated that Se had an interactive effect with Mn. With the Mn/Se ratio increasing, the NBNA scores decreased, and a high level of prenatal Se (≥63.1 µg/L) could block the toxic effects of Mn on early neurodevelopment. The mechanism with which Se protects neonates against neurotoxicity from prenatal Mn exposure remains unclear. Based on the Mn-related oxidative stress and Se-related anti-oxidative stress, we speculate that Se may involve in the protection of brain against excess Mn-induced oxidative stress mainly in the form of antioxidant selenoenzyme. GPx, TRxR and SeP are three important selenoenzymes involved in antioxidant defence. Earlier studies have indicated that subchronic exposure of Mn could result in remarkable reduction in GPx activity of rat striatum [34]. GPx is one of the most important ROS detoxification mechanisms [35]. The clearance of hydrogen peroxide has been reported for cultured astroglial cells, oligodendrocytes, microglial cells and neurons, requiring GPx [36]. And furthermore, TRxR may contribute much more than GPx to brain mitochondrial hydrogen peroxide removal, inhibition of TRxR and GPx can attenuate hydrogen peroxide removal rates in mitochondria by 80% and 25%, respectively [37]. Considering the SeP, apart from its role as Se carrier and supplier to the brain, SeP can be expressed in human astrocytes and involved in the protection of these cells against oxidative stress [38].

It should also be noted that when the cord serum Se was lower (<63.1 µg/L), the protective effects of Se against higher Mn exposure reduced. It is estimated that 0.5–1 billion people in the world are directly affected by Se deficiency [39]. Se is increasingly being considered as a nutraceutical component and many pregnant women take the mineral supplement routinely. As an antioxidant, Se may be considered an alternative therapeutic treatment of Mn neurotoxicity. The supplementation of Se to against the neurotoxicity caused by Mn should be considered during pregnancy, especially in areas with low natural Se.

Our study has some limitations. First, dietary Mn and Se in this study were not investigated. Second, the long-term follow-up investigation was not conducted to explore the interaction of Mn and Se. In addition, although we have adjusted for some potential confounders, in the possibility of residual confounding cannot be ruled out.

## Conclusions

Se has protective effect for neonates against neurotoxicity from prenatal Mn exposure. Se supplementation should be considered during pregnancy, especially in areas with low natural Se. Additional researches are needed to further demonstrate the interaction between Se and Mn.
